# Admission Systemic Immune-Inflammation Index Is Associated with Cognitive Performance and Handgrip Strength Three Months After First-Ever Acute Ischaemic Stroke: A Prospective Cohort Study

**DOI:** 10.3390/brainsci16090948

**Published:** 2026-09-05

**Authors:** Nazemin Gürsoy Karaman, Emine Koç, Buse Çağın

**Affiliations:** 1 Department of Physiology, Faculty of Medicine, Near East University, Near East Boulevard, Nicosia 99138, Cyprus; emine.koc@neu.edu.tr; 2 Department of Neurology, Dr. Burhan Nalbantoğlu State Hospital, Nicosia 99010, Cyprus; busecagin@gmail.com

**Keywords:** acute ischaemic stroke, systemic immune-inflammation index, Montreal Cognitive Assessment, cognitive performance, handgrip strength, physical performance, neurorehabilitation

## Abstract

Background/Objectives: Admission systemic immune-inflammation index (SII) has been associated with post-stroke cognition, but its concurrent relationships with cognitive and physical performance remain less clear. We examined admission SII in relation to three-month Montreal Cognitive Assessment (MoCA) scores and handgrip strength after first-ever acute ischaemic stroke. Methods: This single-centre prospective cohort enrolled 185 patients; 149 completed both three-month assessments and were analysed. Continuous MoCA was the primary outcome, and handgrip strength the secondary outcome. Associations were examined with hierarchical linear models, HC3 robust standard errors, and restricted cubic splines. Results: SII correlated inversely with MoCA (ρ = −0.668; *p* < 0.001) and handgrip strength (ρ = −0.481; *p* < 0.001). After accounting for measured covariates, a twofold increase in SII was associated, on average, across the observed distribution, with a 2.69-point-lower MoCA score (95% CI: −3.52 to −1.86) and 2.20 kgf lower handgrip strength (95% CI: −3.40 to −1.01). Adding ln(SII) to the clinical model increased unadjusted *R*^2^ by 0.209. The adjusted SII–MoCA association was monotonic, inverse, and nonlinear (*p* = 0.002). Conclusions: Higher admission SII was associated with lower three-month cognitive and physical performance in this selected cohort. External validation is required before clinical use.

## 1. Introduction

Acute ischaemic stroke (AIS) is one of the leading causes of permanent neurological disability [1]. Epidemiological data reported from northern Cyprus also confirm the burden of stroke in the area and the importance of vascular risk factors [2]. Cognitive and motor impairments frequently co-occur following a stroke; they can adversely affect independent living, participation in rehabilitation, and quality of life [1]. Biomarkers that are readily available at the time of presentation and are associated with subsequent cognitive or physical function may facilitate the early identification of patients requiring closer monitoring and intensive rehabilitation.

Inflammation is one of the key components of the pathophysiology of ischaemic stroke. Cerebral ischaemia triggers peripheral and central immune responses involving circulating blood cells, cytokines, chemokines and endothelial interactions. Whilst this response plays a role in tissue clearance and repair, when it is excessive or persistent, it can disrupt the blood–brain barrier, exacerbate neuronal damage and limit recovery [3,4,5]. Blood-derived inflammatory indices are therefore being investigated in relation to stroke severity, functional outcome and post-stroke cognitive impairment [6,7,8,9,10,11,12].

The SII, derived from neutrophil, platelet, and lymphocyte counts, integrates inflammation, immune regulation, and thrombo-inflammation into a routine composite measure. Higher SII levels have been associated with greater stroke severity, poorer functional outcomes, and post-stroke cognitive impairment [6,8,9,10,12]. Bao et al. and Cheng et al. reported associations between admission SII and cognitive outcomes at three months in prospective cohorts [6,10]. A recent multicentre longitudinal study further linked acute systemic inflammation to adverse cognitive trajectories [13], whereas a systematic review and meta-analysis found particularly strong evidence for lymphocyte-related indices such as NLR and PLR [14]. Thus, the SII–cognition association is not a new observation. A remaining question is whether the same admission marker relates concurrently to continuous cognitive performance and maximum handgrip performance at a common follow-up point. Because SII has been associated with acute neurological severity [8,9,10,12], and NIHSS is an established measure of stroke severity [15], NIHSS was considered as a distinct adjustment domain.

Post-stroke outcomes extend beyond cognition. Handgrip strength is a simple and reproducible measure of muscle function and physical performance [16]. Lower handgrip strength is associated with adverse cardiovascular and stroke-related outcomes [17,18], and grip performance has been linked to cognitive recovery and broader rehabilitation outcomes after stroke [19,20,21,22]. Evidence remains limited, however, on the concurrent relationships of a single-admission SII measurement with continuous MoCA scores and continuous handgrip strength at the same three-month time point in patients with first-ever AIS. Examining these outcomes within one cohort permits a parallel assessment of complementary cognitive and physical domains.

This study examined the associations of admission SII with cognitive and physical performance three months after AIS. The primary hypothesis, specified before outcome analysis, was that higher SII would be associated with a lower continuous MoCA score. Handgrip strength was defined as a secondary outcome representing physical performance. We expected both associations to remain after accounting for demographic characteristics, vascular comorbidities, stroke subtype, and admission NIHSS. Secondary objectives were to describe the incremental explanatory contribution of SII to the cognitive model relative to NLR and PLR, examine the dose–response shape, and assess the robustness of the principal findings. Binary classifications and data-derived thresholds were confined to exploratory supplementary analyses.

## 2. Materials and Methods

### 2.1. Study Design and Ethics

This single-centre, prospective longitudinal observational cohort study enrolled patients admitted with acute ischaemic stroke between 1 June and 30 December 2025 and included a three-month follow-up assessment. The study was conducted in accordance with the Declaration of Helsinki and Good Clinical Practice. The Scientific Research and Publication Ethics Committee approved the protocol on 15 May 2025 (YTK.1.01 EK15/25). All participants received study information and provided written informed consent before enrolment. Reporting followed the STROBE statement [23]. Because the study assigned no intervention, it was not registered as a clinical trial.

### 2.2. Participants and Data Collection

During the study period, 295 patients with AIS presenting to the Department of Neurology at Dr. Burhan Nalbantoğlu State Hospital were screened for eligibility before the age criterion was applied.

**Figure 1:**
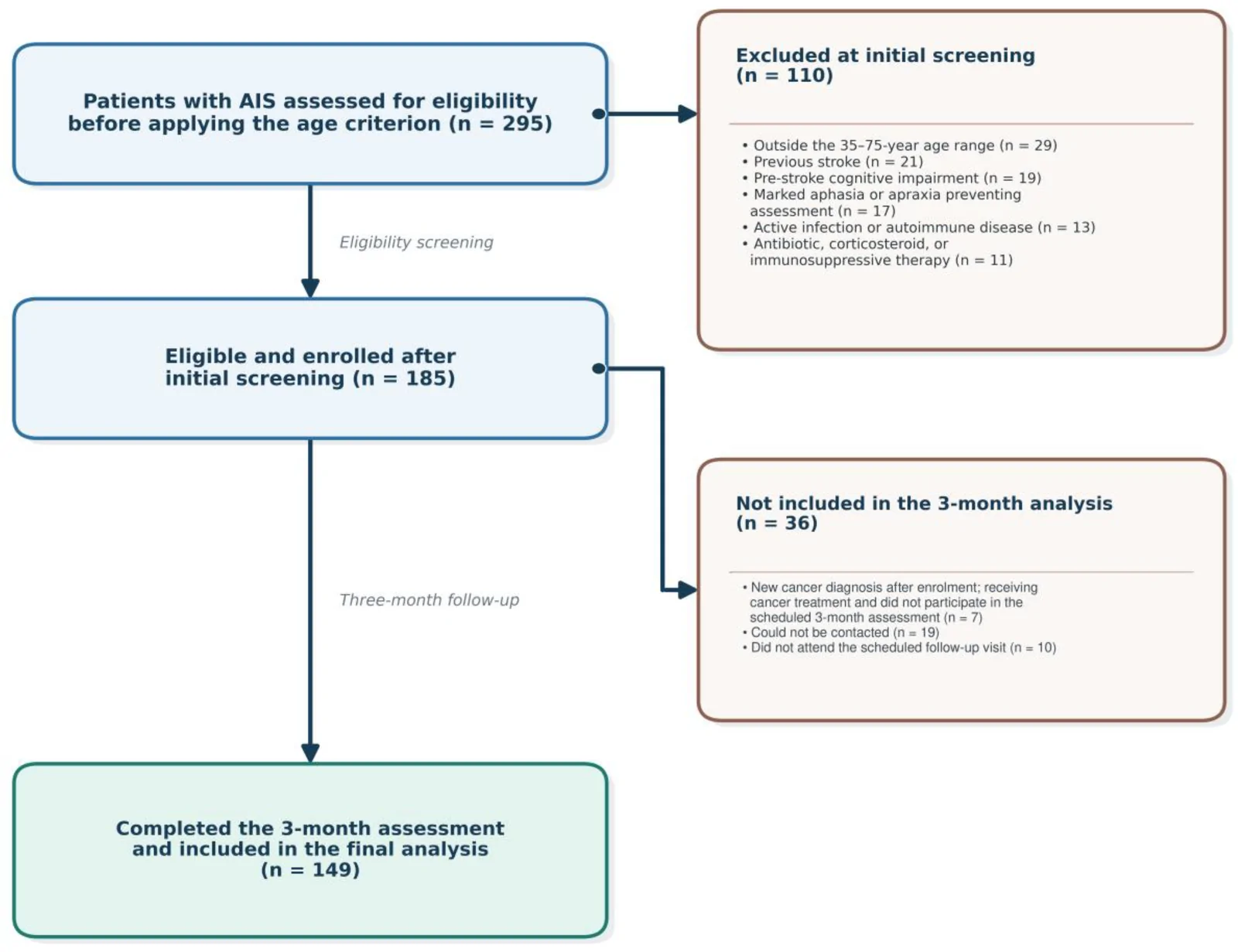
Participant flow from eligibility screening through to the three-month assessment and the final analytical cohort. AIS: acute ischaemic stroke.

Inclusion criteria were CT-confirmed first-ever AIS and age 35–75 years. The 35–75-year range was pre-specified in the study protocol and applied before enrolment. It was a design-stage restriction intended to define a more age-homogeneous cohort. The lower boundary was selected to limit heterogeneity associated with very-young stroke mechanisms, whereas the upper boundary was an operational protocol choice intended to limit age-related variation in inflammatory indices, cognitive performance, and handgrip strength. Neither boundary was selected after inspection of SII or outcome data, or considered a biologically validated cut-off, and age was retained as a continuous covariate in all adjusted models. Initial exclusion criteria were age outside the pre-specified range (*n* = 29), history of prior stroke (*n* = 21), pre-stroke cognitive impairment (*n* = 19), marked aphasia or apraxia-preventing assessment (*n* = 17), active infection or autoimmune disease (*n* = 13), and antibiotic, corticosteroid, or immunosuppressive therapy (*n* = 11). These criteria excluded 110 patients; 185 eligible patients provided written informed consent. Pre-stroke cognitive impairment was identified from documented clinical history in the medical record or cognitive decline before the index stroke reported by a close relative; a standardised retrospective premorbid cognitive instrument was not available. Medication records, medical history, and laboratory results were used to assess active infection and autoimmune disease. Seven participants received a new diagnosis of cancer after enrolment but before the scheduled three-month assessment. They were undergoing cancer treatment and did not participate in the scheduled follow-up visit; consequently, MoCA and handgrip-strength assessments were not performed. Of the remaining participants, 19 could not be contacted and 10 did not attend the scheduled follow-up visit. The final analysis included 149 participants (Figure 1).

Patients were screened consecutively throughout the pre-specified study period. Sample size was determined by the number of consecutive patients who met the eligibility criteria; no post hoc power analysis was performed. Demographic characteristics, clinical history, lifestyle variables, anthropometric measurements, admission laboratory values, ischaemic stroke subtype, and NIHSS score were recorded. NIHSS was assessed within 30 min of admission. Stroke subtype was classified as small-vessel disease, large-artery atherosclerosis, cardioembolic stroke, or other according to the current TOAST-based coding system. A neurologist who was blinded to SII results made this classification using CT, MRI, Holter monitoring, electrocardiography, and echocardiography findings. MoCA and handgrip strength were assessed three months after stroke.

### 2.3. Laboratory Assessment and Inflammatory Indices

The admission blood sample used to calculate SII, NLR, and PLR was obtained within 24 h of hospital admission and before any intravenous thrombolysis or mechanical thrombectomy. The exact interval from symptom onset to sampling was not recorded as a continuous variable. A complete blood count was performed using an automated analyser (XN-1000, Sysmex Corporation, Kobe, Japan). The SII was calculated by dividing the product of the platelet and neutrophil counts by the lymphocyte count. For the NLR, the neutrophil count was divided by the lymphocyte count; for the PLR, the platelet count was divided by the lymphocyte count. All three indices were recalculated from the raw blood cell counts prior to analysis.

### 2.4. Three-Month Dual-Domain Outcome Assessment

Three-month MoCA and handgrip-strength assessments began after day 90 and were completed between days 90 and 100 after stroke onset. Patients were contacted by telephone to arrange the visit, and all completed assessments were conducted during scheduled outpatient follow-up. The same trained researcher conducted all assessments according to a standardised protocol and was blinded to admission SII and other laboratory results. Baseline data-collection forms and admission records were not available to the assessor during the visit. Complete blinding to clinically apparent participant characteristics or information disclosed during the assessment could not be guaranteed. Information on the type, frequency, duration, and intensity of motor or cognitive rehabilitation received during the three-month follow-up period was not systematically collected.

Continuous MoCA score was the primary measure of cognitive performance; continuous handgrip strength was the secondary measure of maximum handgrip performance across both hands. The two complementary outcomes were analysed in separate models, and no data-derived composite endpoint was created.

Cognitive performance was measured at the three-month mark using the Montreal Cognitive Assessment (MoCA). For participants classified in the study records as having completed primary or secondary school, the duration of education was considered to be ≤12 years. For participants in this category whose raw MoCA score was below 30, one point was added in accordance with the standard educational adjustment. No educational correction was applied to university graduates. The MoCA was developed and validated as a brief cognitive screening instrument [24], and Turkish studies have supported its cultural adaptation and discriminative validity [25,26]. The primary outcome was the continuous education-adjusted MoCA score. Binary definitions were used only in exploratory sub-analyses.

Handgrip strength was measured bilaterally with a BIOPAC hand dynamometer and BIOPAC Student Lab software (version 3.7.6; BIOPAC Systems, Inc., Goleta, CA, USA). The device was calibrated before each assessment. Participants were seated with the shoulder adducted and neutral, the elbow flexed to 90°, the forearm neutral, and the wrist extended to 0–30°. After a warm-up, three maximal efforts were recorded from each hand with 30 s of rest between trials. The highest value recorded across the six trials was retained as the participant-level outcome. Results are reported in kilogram-force (kgf; 1 kgf = 9.80665 N). Hand dominance, paretic side, and the laterality of the maximum value selected for analysis were not recorded. Accordingly, the outcome represents maximum handgrip performance across both hands rather than paretic-limb function or side-specific recovery.

### 2.5. Statistical Analysis

Continuous variables are summarised as median and interquartile range (IQR), and categorical variables as frequency and percentage. Bivariate associations were assessed with Spearman’s rank correlation coefficient. Right-skewed SII, NLR, and PLR values were natural-log transformed before modelling. The raw distribution of the primary association was displayed using individual observations, a descriptive linear trend, and a 95% confidence band; the figure reports Spearman’s rank correlation.

The primary SII–MoCA hypothesis was specified at study inception before outcome analysis. The core adjustment set was defined from clinical reasoning and the literature before the main analyses of the expanded dataset; selection was not outcome-driven. Models included age, sex, body mass index (BMI), education, hypertension, diabetes mellitus, smoking status, ischaemic stroke subtype (TOAST; reference category: small-vessel disease), and NIHSS. Education was entered as a three-level categorical variable, with primary school as the reference and indicator terms for secondary school and university. Hierarchical linear models were fitted in three stages: Model 1 was unadjusted; Model 2 included the demographic, anthropometric, vascular, and stroke-subtype covariates; and Model 3 additionally included admission NIHSS. Within each block, all variables assigned to that block were entered simultaneously, and no automatic variable-selection procedure, data-driven subgroup screening, or interaction testing was used. NIHSS was introduced in the final block because it represents the acute neurological severity of the index stroke, a conceptually distinct domain from the background characteristics entered in Model 2. This ordering made the change in the ln(SII) estimate after additional adjustment for stroke severity explicit; it was not used to select variables. MoCA and handgrip strength were modelled separately. Because both outcomes were measured at the same follow-up point, no mediation analysis was undertaken.

The adjustment set was selected to represent demographic, socioeconomic, anthropometric, vascular-risk, and index-stroke domains while limiting model complexity in this cohort. Coronary artery disease and hypercholesterolaemia were collected for cohort description but were not included because the core model already contained hypertension, diabetes mellitus, smoking status, and stroke subtype as indicators of vascular burden. Alcohol use was recorded only as a binary variable, without information on amount, frequency, or drinking pattern, and was therefore considered insufficiently characterised for confounding adjustment. Employment status was not included because education was the selected socioeconomic covariate. No collected variable was screened into or out of the model on the basis of its univariable association with an outcome.

Regression confidence intervals and *p*-values were calculated using heteroscedasticity-consistent HC3 robust standard errors. This approach protects coefficient inference against heteroscedasticity but does not correct a mis-specified mean function or nonlinearity. The ln(SII) coefficient was multiplied by ln(2) to estimate the mean outcome difference associated with a twofold increase in SII under the log-linear model. Multicollinearity was assessed for all terms using variance inflation factors (VIFs). Residual, normal Q–Q, and Cook’s distance plots were examined. Potentially influential observations were retained in the primary analysis; a sensitivity analysis excluded observations above the screening threshold, Cook’s distance > 4/*n*. To compare incremental explanatory contributions, ln(SII), ln(NLR), and ln(PLR) were added separately to the same clinical baseline model and were not entered together. Comparisons used the change in ordinary, unadjusted *R*^2^ (Δ*R*^2^), partial *R*^2^, small-sample corrected Akaike information criterion (*AICc*), HC3 confidence intervals, and optimism-corrected *R*^2^ from 1000 participant-level bootstrap resamples. *AICc* was calculated as AIC + [2K(K + 1)/(*n* – K − 1)], where *n* is the sample size and K is the number of estimated parameters, including regression coefficients and residual variance in Gaussian linear models. AIC was obtained with stats::AIC(), and K from the degrees of freedom returned by stats::logLik(). Model fit was summarised using adjusted *R*^2^. For the incremental comparisons, Δ*R*^2^ and partial *R*^2^ were calculated from ordinary, unadjusted *R*^2^ values. To distinguish these measures clearly, the unadjusted *R*^2^ of the common clinical baseline model and the unadjusted and adjusted *R*^2^ values of each marker-augmented model were reported side by side. Because no direct test of superiority was performed, between-marker model metrics were interpreted descriptively.

The log-linear assumption for SII and the primary outcome was examined using a restricted cubic spline with three knots at the 10th, 50th, and 90th percentiles of ln(SII) [27]. The model was fitted on the ln(SII) scale; the horizontal axis of the spline plot was back-transformed to raw SII for interpretation. For visualisation, spline predictions were displayed from the 5th to the 95th percentile of observed SII (191.3–2647.5), while rug marks showed all 149 observations. The overall association (2 degrees of freedom) and nonlinear component (1 degree of freedom) were tested separately with model-based partial F-tests from the rms::ols model. HC3 correction was not applied to spline tests and was used only for coefficients in log-linear models. When nonlinearity was supported, the spline determined the interpretation of association shape; the doubling coefficient was retained as an average summary across the observed SII range. To quantify the nonlinear association, adjusted MoCA estimates at the 25th, 50th, and 75th SII percentiles and model-based pairwise contrasts were derived from the fully adjusted spline with 95% confidence intervals. Their *p*-values were not adjusted for multiple comparisons and were used only to aid interpretation of the nonlinear association.

To examine whether the findings were influenced by treating MoCA as a continuous outcome, we repeated the fully adjusted analysis using a cumulative logit proportional-odds model. All 21 observed MoCA scores (range, 9–29) were retained as distinct ordered levels rather than collapsed into arbitrarily defined categories. The covariate structure was identical to that of the fully adjusted linear model and included age, sex, BMI, education, hypertension, diabetes mellitus, smoking status, stroke subtype, and admission NIHSS. The ordinal coefficient for ln(SII) was converted to a common odds ratio per twofold increase in SII using exp[β × ln(2)]. Model convergence and estimation warnings were examined. Linear regression remained the primary approach because the target estimand was the adjusted mean difference on the original MoCA-point scale; the ordinal model served as a sensitivity analysis addressing the bounded and ordered nature of the outcome. The ordinal analysis addressed the measurement scale of MoCA, whereas the restricted cubic spline analysis separately examined the functional form of the SII–MoCA association.

Continuous MoCA score was the primary outcome. Supplementary ROC analyses examined cognitive impairment defined as MoCA < 22 and MoCA < 26 and low handgrip strength defined by sex-specific EWGSOP2 thresholds (< 27 kgf for men and < 16 kgf for women) [28,29]. In univariable analyses, AUC and the Youden threshold were calculated on the raw SII scale; because both depend on ranking, they are unchanged by a monotonic log transformation. The parsimonious clinical model comprised age, sex, and admission NIHSS, and ln(SII) was added to form the extended model. Because sex was both a predictor and a component of the sex-specific low-grip definition, a sensitivity analysis omitted sex from both low-grip models: the clinical model included age and admission NIHSS, and the extended model additionally included ln(SII). This analysis estimated discrimination without directly including the variable used to set the outcome threshold. Paired AUCs were compared using DeLong’s test. Multivariable AUCs in Table S1 are apparent, in-sample estimates. The extended model for MoCA < 22 underwent participant-level bootstrap internal validation. All thresholds were derived and evaluated in this cohort and were not regarded as clinically validated cut-offs.

The participant was the sampling unit in all bootstrap analyses. Optimism-corrected *R*^2^ and AUC, calibration slope and intercept, and Brier score were estimated from 1000 resamples using rms::validate(). All tests were two-sided, with *p* < 0.05 denoting statistical significance. Analyses were conducted in R version 4.5.0 (R Foundation for Statistical Computing, Vienna, Austria) [30] using rms (version 8.1.1; ols, lrm, orm, rcs, datadist, Predict, contrast, validate, anova), sandwich (version 3.1.2; vcovHC; type = “HC3”), lmtest (version 0.9.40; coeftest, coefci), car (version 3.1.5; vif), pROC (version 1.19.0.1; roc, ci.auc, coords, roc.test [DeLong]), stats (version 4.5.0; AIC, logLik), ggplot2 (version 4.0.3), and knitr (version 1.51).

Baseline characteristics of the 149 participants in the analytical cohort were compared with those of all 36 enrolled participants who were not included in the three-month analyses, including the seven participants receiving cancer treatment. Continuous variables were compared with the Wilcoxon rank-sum test and sex with Fisher’s exact test. Standardised mean differences (SMDs) provided a sample-size-independent measure of balance. For continuous variables, the SMD was the difference between group means divided by the pooled standard deviation calculated from the mean of the two group variances; the corresponding Bernoulli variance was used for female sex. Because SII was right-skewed and the primary models used ln(SII), baseline balance was assessed principally on the ln(SII) scale; the raw-SII SMD was also reported as sensitivity information. SMDs were interpreted descriptively rather than as significance tests. As an attrition sensitivity analysis, stabilised inverse-probability-of-observation weights were estimated in the full enrolled cohort from a logistic model of three-month outcome availability including age, sex, admission NIHSS, and ln(SII), the baseline variables available for all 185 participants. The same fully adjusted MoCA and handgrip-strength models were then refitted among the 149 participants with observed outcomes using these weights and HC3 robust standard errors. Weight distribution, effective sample size, and covariate balance were assessed. Robustness was examined by truncating weights at the 1st and 99th percentiles and by a 2000-resample participant-level nonparametric bootstrap in which both the observation model and weighted outcome models were refitted in each resample.

### 2.6. Data Quality Assurance

Before analysis, all records were checked against source patient files and identified data-entry errors were corrected. Exposure, covariate, and outcome data were complete for all 149 participants in the analytical cohort, and there were no duplicate records. Recorded SII values were fully consistent with values recalculated from raw platelet, neutrophil, and lymphocyte counts. Primary analyses used complete cases; no missing-data imputation was performed.

## 3. Results

### 3.1. Participant Characteristics and Data Integrity

Before application of the age criterion, 295 patients with AIS were screened. Initial screening excluded 110 patients: age outside 35–75 years (*n* = 29), previous stroke (*n* = 21), pre-stroke cognitive impairment (*n* = 19), marked aphasia or apraxia-preventing assessment (*n* = 17), active infection or autoimmune disease (*n* = 13), and antibiotic, corticosteroid, or immunosuppressive therapy (*n* = 11). The remaining 185 patients were enrolled. During follow-up, seven participants received a new cancer diagnosis after enrolment but before the scheduled three-month assessment. They were undergoing cancer treatment and did not participate in the scheduled follow-up visit; neither MoCA nor handgrip strength was assessed. Of the remaining participants, 19 could not be contacted and 10 did not attend the scheduled follow-up visit. The final analysis included 149 participants (Figure 1).

The median age was 64.0 years (IQR: 57.0–69.0), and 96 participants (64.4%) were women. Median admission NIHSS was 7.0 (IQR: 6.0–10.0), SII was 589.9 (IQR: 390.5–1023.0), three-month MoCA was 22.0 (IQR: 16.0–24.0), and three-month handgrip strength was 19.6 kgf (IQR: 13.9–24.6). Large-artery atherosclerosis was the most frequent stroke subtype (*n* = 77; 51.7%). Variables used in the analyses were complete.

There was no statistical evidence of baseline differences between the analytical cohort and all 36 participants not included in the three-month analyses in age (*p* = 0.517), female sex (*p* = 0.704), admission NIHSS (*p* = 0.181), or SII distribution (*p* = 0.818). Absolute SMDs for age, female sex, NIHSS, and ln(SII) were 0.153, 0.069, 0.196, and 0.062, respectively; the raw-SII SMD was 0.219 (Table S10). In the attrition-weighted analyses, stabilised weights ranged from 0.897 to 1.246, and the effective sample size was 148.3. After weighting, absolute SMDs for variables in the observation model were below 0.006. A twofold increase in SII was associated with a 2.71-point lower MoCA score (HC3 95% CI: −3.54 to −1.88; *p* < 0.001) and 2.19 kgf lower handgrip strength (HC3 95% CI: −3.40 to −0.98; *p* < 0.001). Participant-level bootstrap confidence intervals were −3.49 to −1.93 for MoCA and −3.27 to −1.05 for handgrip strength. Truncation of weights at the 1st and 99th percentiles did not materially change either estimate. These results were nearly identical to the complete-case estimates. Participant characteristics at admission and three months are summarised in Table 1.

### 3.2. Bivariate Associations

SII correlated positively with admission NIHSS (ρ = 0.538; *p* < 0.001) and inversely with three-month MoCA (ρ = −0.668; *p* < 0.001) and handgrip strength (ρ = −0.481; *p* < 0.001). MoCA and handgrip strength were positively correlated (ρ = 0.563; *p* < 0.001). The primary and clinically relevant Spearman correlations are presented in Table 2. These coefficients were not interpreted as causal or independent of confounding. Raw ln(SII)–MoCA observations and a descriptive linear trend are shown in Figure S3.

### 3.3. Primary Outcome: SII and Three-Month Cognitive Performance

In the unadjusted model, ln(SII) was inversely associated with MoCA (B = −4.519; 95% CI: −5.42 to −3.62; *p* < 0.001). The association remained after accounting for the covariates in Model 2 and after adding NIHSS to the full model. In the full log-linear model, a twofold increase in SII was associated, on average, across the observed distribution, with a 2.69-point-lower MoCA score (95% CI: −3.52 to −1.86; *p* < 0.001). This coefficient summarises the average association across the observed SII range. In the full MoCA model, VIFs for ln(SII) and NIHSS were 1.58 and 1.49, respectively; no material multicollinearity was evident. Stroke subtype remained in the model as a clinically defined covariate.

The proportional-odds sensitivity analysis included all 149 participants and converged without warnings. Higher ln(SII) was associated with lower odds of achieving a higher MoCA score (β = −1.928, SE = 0.297; z = −6.49; *p* < 0.001). For each twofold increase in SII, the common odds ratio for being in a higher MoCA category was 0.263 (95% CI: 0.176–0.393). This finding was directionally consistent with the primary linear-model estimate, which indicated a mean difference of −2.69 MoCA points per twofold increase in SII (95% CI: −3.52 to −1.86). Although the coefficients from the two models represent different estimands and cannot be compared directly, the inverse association remained evident when MoCA was analysed as an ordered outcome.

### 3.4. Secondary Outcome: SII and Three-Month Physical Performance

In the unadjusted model, ln(SII) was associated with lower handgrip strength (B = −3.835; 95% CI: −5.25 to −2.42; *p* < 0.001). The association remained after accounting for demographic and clinical covariates and NIHSS. In the full log-linear model, a twofold increase in SII was associated, on average across the observed distribution, with 2.20 kgf lower handgrip strength (95% CI: −3.40 to −1.01; *p* < 0.001). Confidence intervals and *p*-values are based on HC3 robust standard errors (Table 3).

### 3.5. Incremental Explanatory Contribution to the MoCA Model

The common clinical baseline model for MoCA had an unadjusted *R*^2^ of 0.285 and an *AICc* of 891.9. After ln(SII) was added, the unadjusted *R*^2^ increased to 0.493 and the adjusted *R*^2^ was 0.444, corresponding to Δ*R*^2^ = 0.209 and partial *R*^2^ = 0.292. The model had an *AICc* of 843.0 and an optimism-corrected *R*^2^ of 0.402. When ln(SII), ln(NLR), and ln(PLR) were added separately to the same clinical baseline model, the ln(SII) model had the largest Δ*R*^2^ and the lowest *AICc* (Table 4). Because no direct statistical comparison of marker performance was performed, this ordering was interpreted descriptively.

### 3.6. Shape of the SII–MoCA Association

In the fully adjusted restricted cubic spline model, both the overall association between ln(SII) and MoCA (*p* < 0.001) and the nonlinear component (*p* = 0.002) were supported. These *p*-values came from model-based partial F-tests and were not HC3 robust. The association was monotonic and inverse. Its slope was steeper at lower SII values and flatter at higher values; therefore, a single constant log-linear coefficient did not adequately describe the full shape. The spline characterises the association pattern, whereas the doubling coefficient provides an average summary across the observed range.

**Figure 2:**
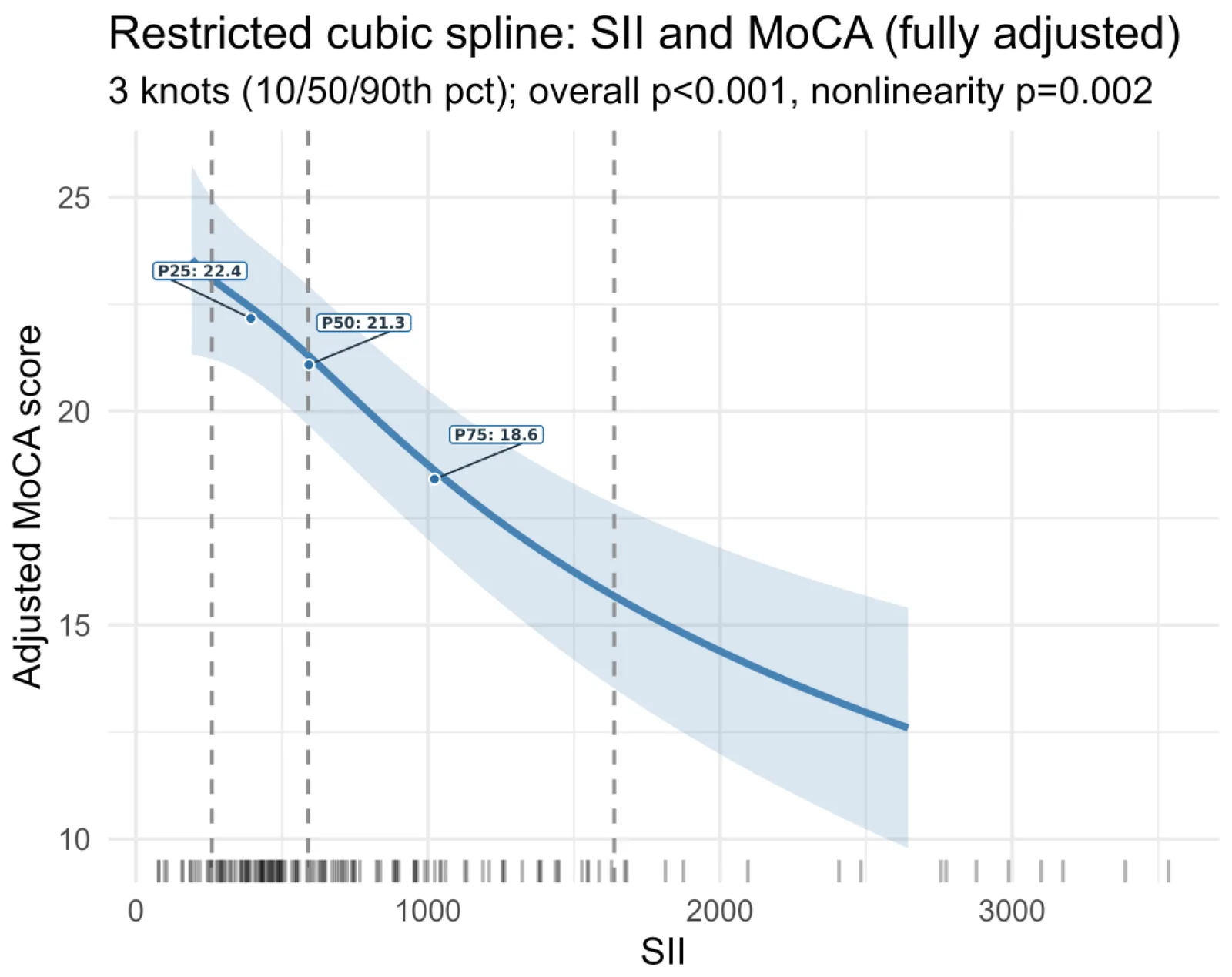
Fully adjusted restricted cubic spline association between admission SII and three-month MoCA. The model used ln(SII), with the horizontal axis back-transformed to the raw SII scale. Knots are located at the 10th, 50th, and 90th percentiles of ln(SII). Shading denotes the 95% confidence interval, rug marks show the complete observed SII distribution, and dashed vertical lines indicate knot locations. Filled circles mark the spline-derived adjusted MoCA estimates at P25 (SII = 390.5; MoCA = 22.4), P50 (SII = 589.9; MoCA = 21.3), and P75 (SII = 1023.0; MoCA = 18.6); their confidence intervals and pairwise contrasts are reported in Table S6. Overall *p* < 0.001; *p* = 0.002 for nonlinearity. Both *p*-values are from model-based partial F-tests without HC3 correction. The prediction curve is displayed from the 5th to the 95th percentile of observed SII (191.3–2647.5).

Spline-derived adjusted MoCA estimates at the 25th, 50th, and 75th SII percentiles were 22.4 (95% CI: 20.8–24.1), 21.3 (95% CI: 19.7–22.9), and 18.6 (95% CI: 16.9–20.4), respectively. Model-based adjusted differences for P50 versus P25, P75 versus P50, and P75 versus P25 were −1.13, −2.69, and −3.82 points, respectively (Table S6). The corresponding *p*-values were unadjusted; these contrasts were not treated as separate confirmatory tests. The fitted spline is shown in Figure 2.

### 3.7. Model Diagnostics and Influential-Observation Sensitivity Analysis

Diagnostic plots showed curvature in the residual pattern of the MoCA model and possible tail deviations in both models. HC3 robust standard errors address coefficient uncertainty under heteroscedasticity but do not correct curvature or a mis-specified mean function. Accordingly, the SII–MoCA shape was interpreted from the spline, and log-linear coefficients were treated as average summaries across the observed range. The largest Cook’s distances were 0.047 for the MoCA model and 0.085 for the handgrip-strength model; no observation exceeded 1. Six and seven observations, respectively, exceeded the screening threshold Cook’s distance > 4/*n*. Excluding these observations changed the ln(SII) coefficient from −3.881 to −4.441 for MoCA and from −3.180 to −3.484 for handgrip strength. Direction was unchanged and magnitude did not attenuate. Primary models retained all participants. Diagnostic plots and sensitivity results are provided in Figures S1 and S2 and Table S8.

### 3.8. Supplementary ROC Analyses

Using the parsimonious clinical model specified in the Methods, the apparent AUC for MoCA < 22 increased from 0.775 (95% CI: 0.701–0.849) to 0.863 (95% CI: 0.802–0.924) after ln(SII) was added (DeLong *p* = 0.001). For MoCA < 26, the corresponding AUCs were 0.609 (95% CI: 0.487–0.731) and 0.808 (95% CI: 0.720–0.896; *p* = 0.011). In bootstrap internal validation of the MoCA < 22 extended model, the apparent AUC of 0.863 was reduced to 0.852 after optimism correction; the calibration slope was 0.934, the calibration intercept was −0.013, and the Brier score was 0.157 (Tables S1 and S2).

For low handgrip strength, the sex-inclusive clinical and extended models had apparent AUCs of 0.877 (95% CI: 0.821–0.933) and 0.899 (95% CI: 0.844–0.953), respectively (DeLong *p* = 0.178). After sex was removed from both models, the corresponding AUCs were 0.642 (95% CI: 0.552–0.732) and 0.729 (95% CI: 0.646–0.811), an apparent increase of 0.087 (*p* = 0.023). These estimates were obtained in the same cohort and were interpreted as supplementary discrimination analyses rather than evidence of clinical prediction performance.

## 4. Discussion

### 4.1. Key Findings and Original Contribution

In this prospective cohort, higher admission SII was associated with lower cognitive performance and maximum handgrip performance three months after AIS. The contribution of this study is not the first demonstration of an SII–cognition association. It is the parallel assessment of a continuous primary MoCA outcome and a secondary continuous handgrip-strength outcome at the same follow-up point within a common adjustment framework. In the full log-linear models, a twofold increase in SII was associated, on average, across the observed range, with a 2.69-point-lower MoCA score and 2.20 kgf lower handgrip strength. Adding SII increased the variance explained by the MoCA model; among the separately fitted biomarker models, the SII model yielded more favourable descriptive fit metrics than the NLR and PLR models. Estimates did not attenuate after potentially influential observations were excluded. The spline further showed that the SII–MoCA association could not be fully summarised by one constant coefficient.

### 4.2. SII and Post-Stroke Cognition

Our findings are consistent with studies linking high baseline SII levels to post-AIS cognitive impairment and adverse outcomes [6,8,9,10,12]. Bao et al. reported an association between SII and cognitive impairment at the three-month mark [6]; Cheng et al. demonstrated a link between baseline SII levels and subsequent cognitive outcomes [10]. In this study, the MoCA was treated as a continuous variable in the main analysis; stroke subtype and NIHSS were included in the same fully adjusted model. The assessment of cognitive performance alongside maximum handgrip performance also adds a different context to the existing evidence. Multicentre longitudinal data suggest that acute systemic inflammation may be associated not only with a single follow-up score but also with adverse cognitive trajectories, and that premorbid cognition and vulnerability may play a role in this relationship [13]. Although meta-analytic evidence supports lymphocyte-related indices such as NLR and PLR, there is high heterogeneity between studies, and the need for external validation persists [14]. In the ordinal sensitivity analysis, which retained each observed MoCA score as a distinct ordered level, higher ln(SII) was likewise associated with lower odds of achieving a higher score. This agreement indicates that the inverse association was not dependent on treating MoCA as a continuous measure; it does not make the linear and ordinal coefficients directly comparable.

Published stroke-rehabilitation work has estimated a MoCA minimally clinically important difference of 1.22 points using an anchor-based approach and 2.15 points using a distribution-based approach [31]. The adjusted 2.69-point difference associated with a twofold increase in SII is therefore of a magnitude that could be clinically relevant at the group level. Direct equivalence to those thresholds would nevertheless be inappropriate: they describe within-person change after rehabilitation, whereas our estimate is an observational between-person difference at one follow-up time point. Baseline MoCA was unavailable, and the spline showed that the association was nonlinear. The estimate should consequently be interpreted as an average difference in three-month performance, not as a clinically important change or an individual treatment effect.

Adjustment for admission NIHSS only modestly attenuated the association between ln(SII) and MoCA. The ln(SII) coefficient changed from −4.095 before NIHSS adjustment to −3.881 after NIHSS was included; the corresponding mean difference per twofold increase in SII changed from −2.84 to −2.69 MoCA points. The unadjusted *R*^2^ increased slightly from 0.491 to 0.493, whereas the adjusted *R*^2^ changed from 0.446 to 0.444 because adjusted *R*^2^ penalises the additional model parameter. Thus, the observed association was not materially explained by admission NIHSS in this cohort. This pattern suggests that SII may reflect information not fully represented by bedside neurological severity, but it does not establish causal independence from stroke severity or support the use of SII as a standalone clinical marker. SII was moderately correlated with NIHSS, and unmeasured features such as infarct volume, lesion location, and reperfusion treatment may still contribute to the association.

The fact that SII integrates complementary cellular processes may provide a biological explanation for the observed association. Neutrophils may contribute to reperfusion injury, protease release and disruption of the blood–brain barrier. Platelet activation may increase microvascular thrombo-inflammation; relative lymphopenia, meanwhile, may reflect a stress response and reduced immune regulation [3,4,5]. These processes may influence the damage around the lesion, neural connections, and the plasticity required for recovery. As these mechanisms were not directly measured in the study, the aforementioned explanations should not be regarded as evidence of causality.

### 4.3. Handgrip Strength and Physical Performance

The adjusted inverse association between SII and maximum handgrip performance across both hands suggests that systemic inflammation may also be related to physical performance after stroke. Alongside immobilisation, catabolism, motor-unit loss, and disuse-related muscle changes, inflammation may contribute to sarcopenia and poorer rehabilitation outcomes [21,22]. Higher SII has also been associated with lower handgrip strength in the general population [32]. In this cohort, the association remained after admission NIHSS was added to the model. However, hand dominance, paretic side, and the laterality of the maximum value selected for analysis were not recorded. Dominance can influence grip strength, and the side affected by stroke may influence motor impairment [33,34]. The result therefore cannot be interpreted as a measure of paretic-limb impairment, inter-limb asymmetry, or side-specific recovery.

### 4.4. Joint Interpretation of the Two Outcome Domains

The positive correlation between MoCA and handgrip strength (ρ = 0.563) suggests that cognitive and physical performance may share clinical determinants. SII was inversely associated with both outcomes, which is compatible with an admission inflammatory burden related to more than one performance domain. These concurrent associations do not establish a shared biological mechanism, causal direction, or mediation by either outcome. Baseline MoCA and handgrip-strength measurements were unavailable, and both outcomes were assessed at three months. The dual-domain design should therefore be interpreted as parallel analyses of complementary outcomes, not as a composite endpoint or mediation model.

### 4.5. Incremental Model Metrics and Nonlinearity

When ln(SII), ln(NLR), and ln(PLR) were added separately to the same clinical baseline model, the SII model yielded a larger Δ*R*^2^ and lower *AICc*. Including platelet count alongside the neutrophil-to-lymphocyte component may carry additional information on cognitive outcome. Nevertheless, the indices are mathematically and biologically related, and their performance was not compared with a direct statistical test. The results do not establish superiority of SII; they show only that the SII model produced more favourable descriptive fit metrics in this cohort.

The steeper spline at lower SII values and flatter segment at higher values indicate that the association is not adequately represented by a single threshold or constant coefficient. MoCA varied along a continuous SII gradient rather than across a sharp boundary. This pattern supports retaining cohort-derived ROC thresholds in the Supplementary Materials rather than presenting them as clinical decision cut-offs.

The revised ROC analyses also show why the low-handgrip results depend on model specification. When sex was included in a model for an outcome defined by sex-specific thresholds, the clinical AUC was already high, and the addition of ln(SII) produced only a small, non-significant increase. Removing sex lowered the clinical AUC and yielded a larger apparent increment after ln(SII) was added. This sensitivity analysis reduces direct definition–model alignment, but it does not establish generalisable prediction: the estimates are in-sample, the comparison was supplementary, and the sex-specific outcome definition remains. No analogous alignment applies to the MoCA outcomes, for which the incremental discrimination associated with ln(SII) remained evident in the parsimonious models.

### 4.6. Strengths and Limitations

Strengths include the prospective design, a primary cognitive hypothesis specified before outcome analysis, and a secondary physical outcome assessed in the same cohort. Data were checked against source records, covariates were selected on clinical grounds before analysis, and stroke subtype and admission NIHSS were retained as clinically defined covariates. HC3 robust inference, bootstrap internal validation, model diagnostics, and influential-observation sensitivity analyses were used. Restricted cubic splines identified an association pattern that a log-linear model alone would have obscured, while the proportional-odds sensitivity analysis addressed the bounded and ordered scale of MoCA. Retaining continuous outcomes for the primary analyses and confining threshold-based ROC analyses to the Supplementary Materials limited information loss and reduced data-driven interpretation.

Several limitations warrant emphasis. This was a single-centre cohort of moderate size, and generalisability may be affected by patient selection and local care pathways. Patients with marked aphasia or apraxia and those with pre-stroke cognitive impairment were excluded; the findings therefore apply to a selected group able to complete MoCA and three-month assessments rather than to the full ischaemic-stroke population. Pre-stroke cognitive impairment was determined from clinical records and informant history rather than a standardised retrospective premorbid cognitive instrument, which may have introduced misclassification. The 35–75-year range was a pre-specified design restriction intended to define a more age-homogeneous cohort. Adults younger than 35 years show a greater contribution of non-traditional stroke mechanisms and risk factors [35], while reference distributions of CBC-derived inflammatory indices [36], normative MoCA performance [37], and handgrip strength [38] vary with age. The upper age boundary was an operational protocol choice rather than an evidence-based biological threshold. Neither 35 nor 75 years should be interpreted as a universally validated cut-off. This restriction limits generalisability to patients younger than 35 years and, particularly, to those older than 75 years.

Among 185 enrolled participants, seven received a new cancer diagnosis after enrolment but before the scheduled three-month assessment. These participants were receiving cancer treatment and did not participate in follow-up. A further 19 could not be contacted and 10 did not attend the scheduled visit. Thus, 36 participants (19.5%) were not included in the complete-case analyses, leaving 149/185 (80.5%) in the analytical cohort. Baseline comparison of the analytical cohort with all 36 non-analysed participants showed only small-to-moderate imbalances, and inverse-probability weighting produced estimates that were nearly identical to the complete-case results. However, the observation model was limited to age, sex, admission NIHSS, and ln(SII), because these were the baseline variables available for all 185 participants. Attrition related to unmeasured factors or to the clinical circumstances surrounding cancer treatment therefore cannot be excluded.

The observational design does not permit causal inference, and unmeasured confounders may remain. Reperfusion therapy, infarct volume and location, lesion side, post-stroke depression, standardised pre-stroke functional status, objective premorbid cognition, premorbid frailty, and baseline grip strength were not recorded. Although the admission sample was obtained within 24 h and before reperfusion treatment, the exact symptom-onset-to-sampling interval was unavailable. In addition, the type, frequency, duration, and intensity of motor or cognitive rehabilitation during follow-up were not systematically collected. Differences in rehabilitation exposure may therefore have contributed to variation in three-month performance and to residual confounding of the observed associations. SII was measured only at presentation; changes in the inflammatory burden over time are unknown. As baseline MoCA and grip-strength measurements were unavailable, cognitive or physical changes could not be calculated. The MoCA is a screening tool and does not replace a comprehensive neuropsychological assessment. Although both hands were assessed, hand dominance, paretic side, and the laterality of the maximum value selected for analysis were not recorded. Because these factors can influence grip performance after stroke [33,34], inter-limb asymmetry and paretic-limb or side-specific recovery could not be examined. Although the adjustment set was defined before analysis, residual confounding by vascular, lifestyle, or socioeconomic characteristics that were not modelled or were incompletely characterised cannot be excluded.

Spline *p*-values for the overall and nonlinear components came from partial F-tests without HC3 correction; inference about association shape should be viewed accordingly. ROC thresholds were derived and evaluated in the same dataset, and the AUCs in Table S1 are apparent, in-sample estimates. Bootstrap correction does not replace independent external validation. Cognitive impairment defined as MoCA < 26 occurred in 128/149 participants (85.9%), leaving only 21 negative cases and limiting the reliability of discrimination estimates. Low handgrip strength was defined using sex-specific thresholds; consequently, including sex in the clinical model created direct definition–model alignment. The lower AUCs obtained after sex was removed showed that the sex-inclusive estimates were partly dependent on this alignment. Although the parsimonious MoCA < 22 extended model showed limited optimism (apparent AUC, 0.863; optimism-corrected AUC, 0.852; calibration slope, 0.934), these internal estimates cannot establish transportability. The ROC analyses therefore remain hypothesis-generating and should not be used as a clinical prediction model or decision threshold.

### 4.7. Clinical and Research Implications

SII can be calculated from a routine complete blood count without additional testing cost, making it a candidate adjunctive marker rather than a substitute for standard clinical assessment. The dual-domain pattern supports further studies that jointly assess cognitive monitoring, maximum handgrip performance, and rehabilitation needs in patients with higher SII. Individual decisions should not rely on a single SII value or on a threshold derived from this cohort. Future multicentre studies should incorporate imaging, reperfusion treatment, depression, and rehabilitation variables and should use repeated SII measurements together with baseline and follow-up cognitive and physical assessments.

## 5. Conclusions

Among selected patients with first-ever AIS, higher admission SII was associated with lower MoCA scores and handgrip strength at three months. The study extends existing SII–cognition evidence by examining continuous cognitive performance and maximum handgrip performance in the same cohort and at the same follow-up point. The inverse cognitive association also remained evident when the observed MoCA scores were analysed as ordered levels. SII increased the explanatory variance of the MoCA model and produced more favourable descriptive fit metrics than separately fitted NLR and PLR models, but these findings neither establish biomarker superiority nor define a clinical threshold. Nonlinearity also limits interpretation based on one constant coefficient. Independent multicentre validation is required before prognostic or clinical use.

## Figures and Tables

**Table 1:** Participant characteristics at admission and three months (*n* = 149).

Characteristics	Value
Age, years	64.0 (57.0–69.0)
Sex—male	53 (35.6%)
Sex—female	96 (64.4%)
Education—primary school	58 (38.9%)
Education—secondary school	63 (42.3%)
Education—university	28 (18.8%)
Employed	44 (29.5%)
Current smoking	56 (37.6%)
Alcohol consumption	47 (31.5%)
BMI, kg/m^2^	26.5 (25.4–28.4)
Hypertension	75 (50.3%)
Diabetes mellitus	67 (45.0%)
Hypercholesterolaemia	67 (45.0%)
Coronary artery disease	44 (29.5%)
Stroke subtype—small-vessel disease	37 (24.8%)
Stroke subtype—large-artery atherosclerosis	77 (51.7%)
Stroke subtype—cardioembolic	19 (12.8%)
Stroke subtype—other	16 (10.7%)
NIHSS score	7.0 (6.0–10.0)
SII	589.9 (390.5–1023.0)
NLR	2.4 (1.7–4.0)
PLR	109.5 (88.7–141.2)
MoCA score at three months	22.0 (16.0–24.0)
Handgrip strength at three months, kgf	19.6 (13.9–24.6)

Data are presented as median (IQR) or *n* (%). BMI: body mass index; NIHSS: National Institutes of Health Stroke Scale; SII: systemic immune-inflammation index; NLR: neutrophil-to-lymphocyte ratio; PLR: platelet-to-lymphocyte ratio; MoCA: Montreal Cognitive Assessment; kgf: kilogram-force.

**Table 2:** Primary and clinically relevant Spearman correlations.

Variable Pair	ρ	*p*
NIHSS–SII	0.538	<0.001
NIHSS–MoCA	−0.377	<0.001
NIHSS–handgrip strength	−0.235	0.004
SII–MoCA	−0.668	<0.001
SII–handgrip strength	−0.481	<0.001
MoCA–handgrip strength	0.563	<0.001

*p*-values are two-sided. SII–MoCA was the primary correlation; the remaining associations are secondary and provide clinical context. No multiple-comparison correction was applied. The exploratory 15-pair correlation matrix is provided in Table S9. Abbreviations are defined in Table 1.

**Table 3:** Hierarchical linear models for SII with HC3 robust inference.

Outcome	Model	ln(SII), B	95% CI	*p*	Adjusted *R*^2^	Mean Difference per Doubling (95% CI)
MoCA	Model 1: unadjusted	−4.519	−5.42 to −3.62	<0.001	0.445	−3.13 (−3.76 to −2.51)
MoCA	Model 2: covariates	−4.095	−5.13 to −3.06	<0.001	0.446	−2.84 (−3.55 to −2.12)
MoCA	Model 3: +NIHSS	−3.881	−5.07 to −2.69	<0.001	0.444	−2.69 (−3.52 to −1.86)
Handgrip strength	Model 1: unadjusted	−3.835	−5.25 to −2.42	<0.001	0.206	−2.66 (−3.64 to −1.68)
Handgrip strength	Model 2: covariates	−3.157	−4.72 to −1.60	<0.001	0.280	−2.19 (−3.27 to −1.11)
Handgrip strength	Model 3: +NIHSS	−3.180	−4.90 to −1.46	<0.001	0.275	−2.20 (−3.40 to −1.01)

Model 2 includes age, sex, BMI, education (reference: primary school; indicator categories: secondary school and university), hypertension, diabetes mellitus, smoking, and stroke subtype. Model 3 additionally includes NIHSS. B coefficients and confidence intervals use HC3 robust standard errors. Differences per doubling are average summaries from the log-linear model across the observed range; handgrip-strength differences are reported in kgf. The SII–MoCA association shape should be interpreted from the spline analysis. Adjusted *R*^2^: coefficient of determination adjusted for model size; CI: confidence interval. *R*^2^ values reported in this table are adjusted *R*^2^ values.

**Table 4:** Incremental explanatory metrics obtained by adding each inflammatory marker separately to the same clinical baseline model for MoCA.

**Panel (A). Model-Fit and Incremental Explanatory Metrics**							
**Marker**	**Baseline *R*^2^**	**Augmented *R*^2^**	**Δ*R*^2^**	**Adjusted *R*^2^**	**Partial *R*^2^**	***AICc***	**Optimism-corrected *R*^2^**
ln(SII)	0.285	0.493	0.209	0.444	0.292	843.0	0.402
ln(NLR)	0.285	0.469	0.184	0.418	0.257	850.1	0.370
ln(PLR)	0.285	0.400	0.116	0.343	0.162	868.1	0.287
**Panel (B). Marker Coefficients with HC3 Robust Inference**							
**Marker**		**HC3 B**		**HC3 95% CI**		***p***	
ln(SII)		−3.881		−5.07 to −2.69		<0.001	
ln(NLR)		−3.980		−5.26 to −2.70		<0.001	
ln(PLR)		−4.524		−6.39 to −2.66		<0.001	

Each marker was added separately to the same clinical baseline model; the markers were not entered together. The baseline model included age, sex, BMI, education (reference: primary school; indicator categories: secondary school and university), hypertension, diabetes mellitus, smoking, stroke subtype, and admission NIHSS. Baseline *R*^2^ and augmented *R*^2^ are ordinary, unadjusted values; Δ*R*^2^ and partial *R*^2^ were calculated from these values. Adjusted *R*^2^ refers to the corresponding marker-augmented model. The *AICc* for the common baseline model was 891.9. Optimism correction was based on 1000 participant-level bootstrap resamples. Between-marker comparisons were descriptive; no direct statistical test of marker superiority was performed.

## Data Availability

The data presented in this study are available from the corresponding author upon reasonable request. The data are not publicly available because of participant privacy and ethics restrictions.

## Supplementary Materials

The following supporting information can be downloaded at: https://www.mdpi.com/article/10.3390/brainsci16090948/s1, Table S1. Exploratory ROC discrimination using the parsimonious clinical model and a sex-excluded low-handgrip sensitivity analysis; Table S2. Participant-level bootstrap internal validation of the age-, sex-, NIHSS-, and ln(SII)-based model for MoCA < 22 (1000 resamples); Table S3. Three-month outcomes across SII tertiles; Table S4. Full coefficients from the MoCA model (HC3 robust inference); Table S5. Full coefficients from the handgrip-strength model (HC3 robust inference); Table S6. Spline-derived adjusted MoCA estimates and pairwise contrasts at SII percentiles (full covariate adjustment); Table S7. Variance inflation factors for all terms in the full model; Figure S1. Regression diagnostics for the full MoCA model; Figure S2. Regression diagnostics for the full handgrip-strength model; Table S8. Influential-observation sensitivity analysis; Figure S3. Raw distribution of ln(SII) and three-month MoCA (*n* = 149); Table S9. Full Spearman correlation matrix for selected continuous variables; Table S10. Baseline characteristics of the analytical cohort and all 36 participants not included in the three-month analyses; Table S11. Proportional-odds ordinal sensitivity analysis for the association between ln(SII) and three-month MoCA. The *p*-values in Table S6 were unadjusted for multiple comparisons and were considered exploratory. Thresholds and apparent AUCs were derived in the same cohort and are hypothesis-generating. Owing to the high outcome prevalence for MoCA < 26 and the definition–model alignment in the sex-inclusive low-handgrip analysis, these results should not be used for clinical decisions.
