# Supplementary material for: Admission Systemic Immune-Inflammation Index Is Associated with Cognitive Performance and Handgrip Strength Three Months After First-Ever Acute Ischaemic Stroke: A Prospective Cohort Study
Source: Brain Sci. 2026 Sep 5;16(9):948. doi: 10.3390/brainsci16090948 (PMC13604118; doi:10.3390/brainsci16090948)
Supplement: Supplementary file 1 [file brainsci-16-00948-s001.zip › brainsci-4521829-supplementary.pdf]

Supplementary Materials

Admission Systemic Immune-Inflammation Index Is Associated with Cognitive Performance and Handgrip Strength Three Months after First-Ever Acute Ischaemic Stroke: A Prospective Cohort Study

All dichotomous and categorical analyses are exploratory. Candidate thresholds were derived and evaluated in the same cohort and should not be used as clinical decision thresholds.

Table S1. Exploratory ROC discrimination using the parsimonious clinical model and a sex-excluded low-handgrip sensitivity analysis.

Outcome / analysis	n (%)	Raw SII AUC (95% CI)	Youden	Clinical AUC (95% CI)	+ln(SII) AUC (95% CI)	DeLong p
MoCA < 22	73 (49%)	0.838 (0.772–0.904)	647	0.775 (0.701–0.849)	0.863 (0.802–0.924)	0.001
MoCA < 26	128 (86%)	0.798 (0.703–0.894)	381	0.609 (0.487–0.731)	0.808 (0.720–0.896)	0.011
Low grip: sex-inclusive	70 (47%)	0.727 (0.644–0.810)	494	0.877 (0.821–0.933)	0.899 (0.844–0.953)	0.178
Low grip: sex-excluded sensitivity	70 (47%)	—	—	0.642 (0.552–0.732)	0.729 (0.646–0.811)	0.023

Univariable SII AUCs and Youden thresholds were calculated on the raw SII scale. The parsimonious clinical model included age, sex, and admission NIHSS; ln(SII) was added to form the extended model. Because low handgrip strength was defined using sex-specific EWGSOP2 thresholds, the sensitivity analysis omitted sex from both models (age + NIHSS versus age + NIHSS + ln[SII]). The raw-SII analysis is unaffected by the predictor set and is therefore shown only once for low handgrip strength. Multivariable AUCs are apparent, in-sample estimates; paired comparisons used DeLong's test. MoCA <22 was the main supplementary binary definition, whereas MoCA <26 was a sensitivity definition. AUC: area under the receiver operating characteristic curve; CI: confidence interval; MoCA: Montreal Cognitive Assessment; SII: systemic immune-inflammation index.

Table S2. Participant-level bootstrap internal validation of the age-, sex-, NIHSS-, and ln(SII)-based model for MoCA <22 (1000 resamples).

Metric	Value
Apparent AUC	0.863
Estimated optimism in AUC	0.011
Optimism-corrected AUC	0.852
Calibration slope	0.934
Calibration intercept	−0.013
Brier score	0.157

The model included age, sex, admission NIHSS, and ln(SII). The resampling unit was the individual participant, and all 1000 resamples were used without convergence warnings. Apparent AUC is the same in-sample estimate reported for the extended MoCA <22 model in Table S1. Optimism-corrected AUC, calibration slope, calibration intercept, and Brier score were calculated with rms::validate(). Internal validation in the same cohort does not replace independent external validation. AUC: area under the receiver operating characteristic curve; MoCA: Montreal Cognitive Assessment; SII: systemic immune-inflammation index.

Table S3. Three-month outcomes across SII tertiles.

Outcome	SII tertile	Median (IQR)	Kruskal-Wallis p	Bonferroni pairwise p (L-M / L-H / M-H)
MoCA	Low	24.0 (22.2-26.0)	<0.001	<0.001 / <0.001 / <0.001
MoCA	Middle	22.0 (20.0-24.0)		
MoCA	High	14.0 (12.0-18.8)		
Handgrip strength	Low	22.9 (18.4-25.4)	<0.001	0.144 / <0.001 / 0.001
Handgrip strength	Middle	21.6 (16.8-23.7)		
Handgrip strength	High	13.8 (11.5-19.4)		

L: low SII; M: middle SII; H: high SII. Values rounded to zero by the software are reported as p < 0.001.

Table S4. Full coefficients from the MoCA model (HC3 robust inference).

Term	B	95% HC3 CI	p
Intercept	53.379	41.65 to 65.10	<0.001
ln(SII)	-3.881	-5.07 to -2.69	<0.001
Age	-0.072	-0.15 to 0.01	0.086
Sex: female	0.744	-0.81 to 2.30	0.346
BMI	-0.167	-0.40 to 0.07	0.161
Education: secondary school	0.742	-0.91 to 2.39	0.376
Education: university	1.332	-0.97 to 3.63	0.255
Hypertension: present	-0.137	-1.60 to 1.33	0.854
Diabetes mellitus: present	0.223	-1.14 to 1.58	0.747
Current smoking: no	0.065	-1.29 to 1.42	0.924
Stroke subtype: large-artery atherosclerosis	0.583	-1.04 to 2.20	0.479
Stroke subtype: cardioembolic	0.905	-1.32 to 3.13	0.422
Stroke subtype: other	0.248	-2.02 to 2.52	0.829
NIHSS	-0.083	-0.29 to 0.13	0.435

Table S5. Full coefficients from the handgrip-strength model (HC3 robust inference).

Term	B	95% HC3 CI	p
Intercept	32.878	17.25 to 48.51	<0.001
ln(SII)	-3.180	-4.90 to -1.46	<0.001
Age	0.022	-0.09 to 0.13	0.684

Term	B	95% HC3 CI	p
Sex: female	1.890	-0.34 to 4.12	0.096
BMI	0.213	-0.14 to 0.56	0.232
Education: secondary school	1.086	-1.25 to 3.42	0.359
Education: university	3.098	0.27 to 5.93	0.032
Hypertension: present	-2.726	-4.90 to -0.55	0.015
Diabetes mellitus: present	-0.384	-2.26 to 1.49	0.687
Current smoking: no	0.537	-1.52 to 2.60	0.607
Stroke subtype: large-artery atherosclerosis	-1.458	-3.81 to 0.89	0.222
Stroke subtype: cardioembolic	-0.904	-4.17 to 2.36	0.585
Stroke subtype: other	-1.438	-5.38 to 2.50	0.472
NIHSS	0.009	-0.29 to 0.31	0.953

Reference categories were male sex, primary-school education, no hypertension, no diabetes mellitus, current smoking, and small-vessel disease. CI: confidence interval.

Table S6. Spline-derived adjusted MoCA estimates and pairwise contrasts at SII percentiles (full covariate adjustment).

Percentile / contrast	SII	Adjusted MoCA or difference (95% CI)	p
P25	390.5	22.4 (20.8–24.1)	—
P50	589.9	21.3 (19.7–22.9)	—
P75	1023.0	18.6 (16.9–20.4)	—
**Pairwise contrasts**			
P50–P25	—	−1.13 (−1.64 to −0.62)	<0.001
P75–P50	—	−2.69 (−3.34 to −2.04)	<0.001
P75–P25	—	−3.82 (−4.78 to −2.86)	<0.001

Estimates and contrasts were derived from the restricted cubic spline model adjusted for age, sex, BMI, education, hypertension, diabetes mellitus, smoking, stroke subtype, and NIHSS. A contrast was defined as the first percentile minus the second percentile. Pairwise p-values were not adjusted for multiple comparisons; the contrasts were used only to aid interpretation of the nonlinear association. Values rounded to zero by the software are reported as p < 0.001. CI: confidence interval; MoCA: Montreal Cognitive Assessment; SII: systemic immune-inflammation index.

Table S7. Variance inflation factors for all terms in the full model.

Term	VIF
ln(SII)	1.58
Age	1.40
Sex	1.24
BMI	1.26
Education	1.29
Hypertension	1.16
Diabetes mellitus	1.12
Smoking	1.15
Stroke subtype	1.07
NIHSS	1.49

The highest VIF was 1.58. For multilevel factors, the square of the adjusted GVIF^(1/(2·df)) is shown.

Figure S1. Regression diagnostics for the full MoCA model.

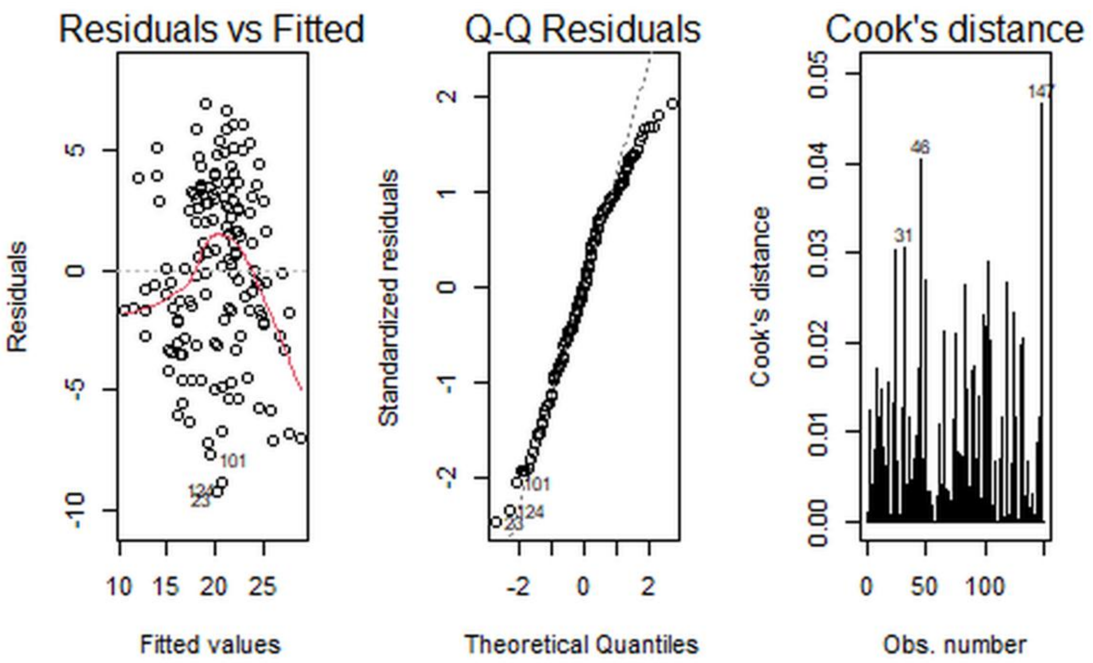

Panels show residuals versus fitted values, the normal Q-Q plot of standardized residuals, and Cook's distance by observation number.

Figure S2. Regression diagnostics for the full handgrip-strength model.

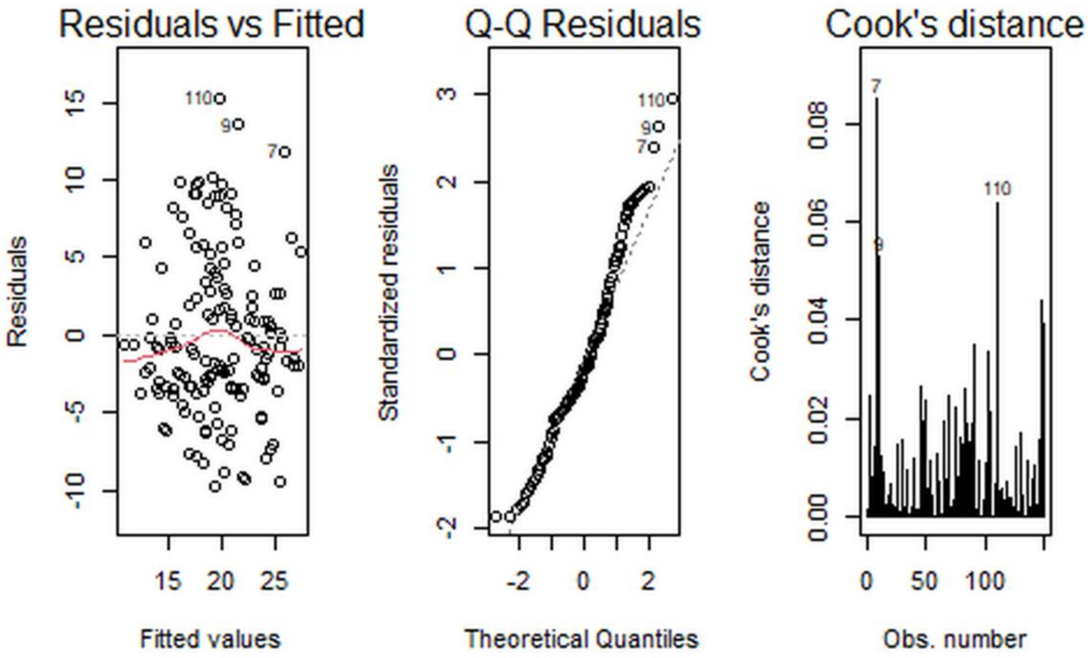

Panels show residuals versus fitted values, the normal Q-Q plot of standardized residuals, and Cook's distance by observation number.

Table S8. Influential-observation sensitivity analysis.

Outcome	Maximum Cook's D	n > 4/n	Cook's D > 1	ln(SII) B: all observations	ln(SII) B: influential observations excluded
MoCA	0.047	6	No	-3.881	-4.441
Handgrip strength	0.085	7	No	-3.180	-3.484

Cook's distance > 4/n was used only as a screening threshold. All participants were retained in the main models; observations were excluded only in the sensitivity analysis.

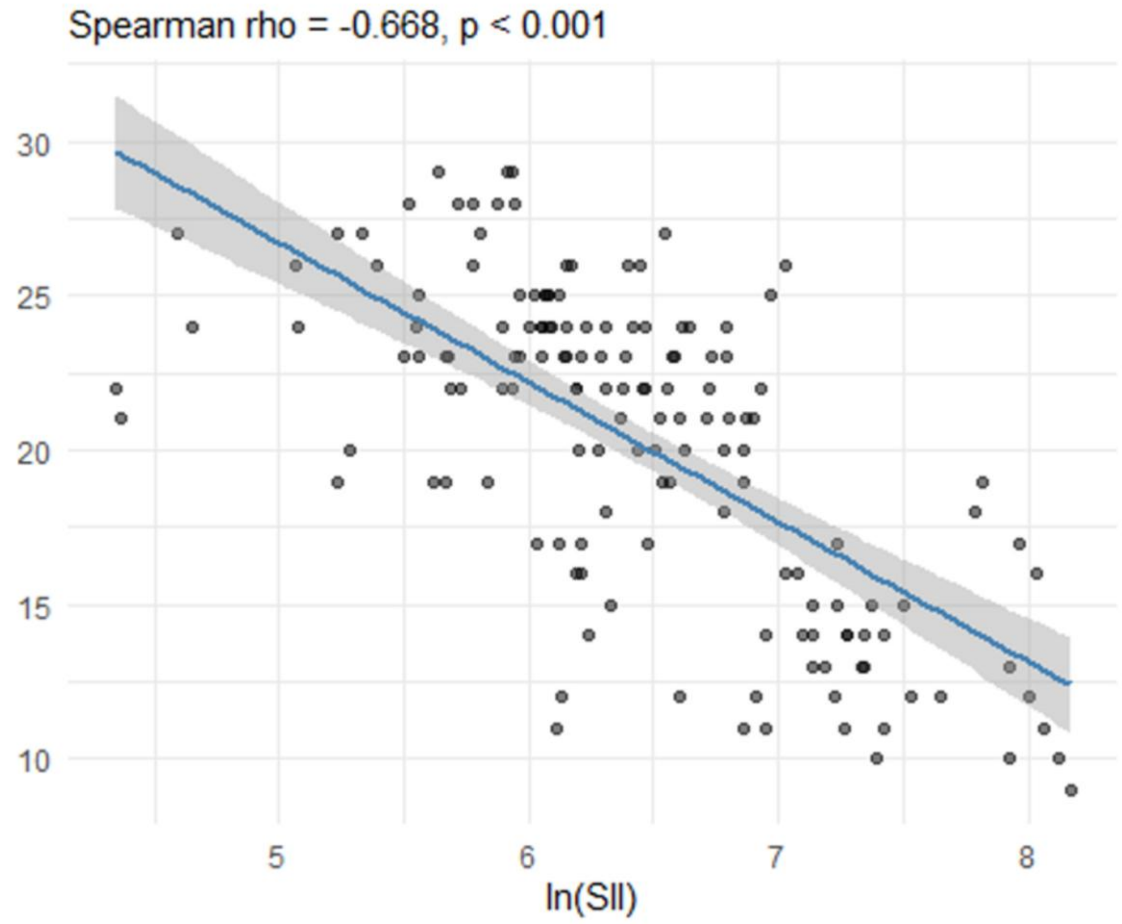

Figure S3. Raw distribution of ln(SII) and three-month MoCA (n = 149). Each point represents one participant. The blue line is a descriptive least-squares linear trend and the grey band is its 95% confidence interval. The displayed rho and p-value are from the Spearman rank correlation (rho = -0.668; p < 0.001). This figure presents the unadjusted distribution; the covariate-adjusted association was examined with a restricted cubic spline in Figure 2 of the main manuscript.

Table S9. Full Spearman correlation matrix for selected continuous variables.

Variable pair	Spearman rho	p
Age – BMI	−0.121	0.142
Age – NIHSS	0.085	0.300
Age – SII	0.208	0.011
Age – MoCA	−0.286	<0.001
Age – handgrip strength	−0.169	0.039
BMI – NIHSS	−0.174	0.034
BMI – SII	−0.071	0.393
BMI – MoCA	−0.010	0.907
BMI – handgrip strength	0.137	0.096
NIHSS – SII	0.538	<0.001
NIHSS – MoCA	−0.377	<0.001
NIHSS – handgrip strength	−0.235	0.004
SII – MoCA	−0.668	<0.001
SII – handgrip strength	−0.481	<0.001
MoCA – handgrip strength	0.563	<0.001

SII-MoCA was the prespecified primary correlation. The remaining associations are secondary or exploratory descriptions that provide clinical context; no adjustment for multiple comparisons was applied. All p-values are two-sided. BMI: body mass index; NIHSS: National Institutes of Health Stroke Scale; SII: systemic immune-inflammation index; MoCA: Montreal Cognitive Assessment.

Table S10. Baseline characteristics of the analytical cohort and all 36 participants not included in the three-month analyses.

Variable	Analytical cohort (n = 149)	All participants not included in the three-month analyses (n = 36)	p	|SMD|
Age, years	64.0 (57.0–69.0)	60.5 (54.8–69.0)	0.517	0.153
Female sex, n (%)	96 (64.4%)	22 (61.1%)	0.704	0.069
Admission NIHSS	7.0 (6.0–10.0)	8.5 (6.0–12.0)	0.181	0.196
SII, raw scale	589.9 (390.5–1023.0)	635.6 (376.5–937.1)	0.818	0.219
ln(SII)	6.380 (5.967–6.930)	6.454 (5.930–6.843)	0.818	0.062

Continuous variables are presented as median (IQR), and sex as n (%). Continuous variables were compared with the Wilcoxon rank-sum test and sex with Fisher's exact test. SMD: standardised mean difference; IQR: interquartile range; NIHSS: National Institutes of Health Stroke Scale; SII: systemic immune-inflammation index. Absolute SMD values are reported. For continuous variables, the SMD was calculated as the difference between group means divided by the standard deviation derived from the mean of the two group variances; the corresponding Bernoulli variance was used for female sex. Because SII was right-skewed and log-transformed in the main models, baseline balance was assessed primarily on the ln(SII) scale; the raw SII scale is also shown. SMDs are descriptive. The comparison includes all 36 non-analysed participants, including the seven who received a new cancer diagnosis after enrolment. Because the analysis is limited to available baseline variables, it cannot exclude selective attrition.

Table S11. Proportional-odds ordinal sensitivity analysis for the association between ln(SII) and three-month MoCA.

Metric	Result
Analysis	Fully adjusted cumulative logit proportional-odds model
Participants	149
Observed MoCA levels	21 distinct scores (range, 9–29)
ln(SII), β (SE)	−1.928 (0.297)
z	−6.49
p	8.3×10^{-11}
Common OR per twofold increase in SII (95% CI)	0.263 (0.176–0.393)
Convergence	Converged without warnings

MoCA was analysed using all 21 observed scores as ordered outcome levels; no cut-off or artificial grouping was introduced. The model adjusted for age, sex, BMI, education, hypertension, diabetes mellitus, smoking status, stroke subtype, and admission NIHSS. The odds ratio represents the common odds of being in a higher MoCA category per twofold increase in SII. The model was fitted using rms::orm and converged without warnings. Linear regression remained the primary analysis because it estimates adjusted mean differences on the original MoCA-point scale; the ordinal model was used to assess sensitivity to the treatment of MoCA as a continuous outcome. CI: confidence interval; MoCA: Montreal Cognitive Assessment; OR: odds ratio; SII: systemic immune-inflammation index.
